# Knowledge and management of traumatic dental injuries among schoolteachers in Hungary: a cross-sectional study with educational intervention

**DOI:** 10.1007/s40368-024-00862-1

**Published:** 2024-02-05

**Authors:** M. Fittler, A. Fittler, T. Dergez, A. Radácsi, K. Katona, B. Sándor, I. Szántó

**Affiliations:** 1 Department of Dentistry, Oral and Maxillofacial Surgery, Medical School and Clinical Center, University of Pécs, Tüzér Str. 1, Pécs, 7623 Hungary; 2 Department of Pharmaceutics, Faculty of Pharmacy, University of Pécs, Pécs, Hungary; 3 Institute of Bioanalysis, Medical School, University of Pécs, Pécs, Hungary

**Keywords:** Dental injuries, Dental trauma, First-aid, Teacher awareness, Dental trauma, First-aid, Dental injuries

## Abstract

**Purpose:**

This study aimed to evaluate the knowledge of Hungarian schoolteachers in the management of dental trauma injuries (TDI) of children between the ages of 3 and 18 and to illustrate a brief educational intervention on TDI management.

**Methods:**

A 15-item questionnaire on dental injuries was distributed in our observational cross-sectional study to 2720 Hungarian educational institutions to explore and evaluate teachers’ knowledge in January 2019. Two years later, targeted information material was made accessible regarding TDI management. In the second post-intervention phase of the study, educator knowledge was re-evaluated using the same questionnaire. Statistical analysis (Mann–Whitney and Chi-square tests) was performed using IBM SPSS Statistics 28.

**Results:**

A total of 1426 answers were collected in the initial survey. Although more than half (51.9%) of the respondents previously witnessed TDIs, 86.5% still did not perceive themselves as adequately informed regarding TDI management. Most teachers submitted appropriate responses to the indicator questions relating to the urgency of referral to dental professionals (71.8%), immediate contact with parents (79.0%) or dentists (13.0%), and the solution for avulsed teeth (81.3%). However, only every second (56.2%) educator responded correctly regarding the proper cleaning method. Following accessibility to our educational material, 622 respondents completed the post-intervention questionnaire in the second phase of the study. The percentage of appropriate responses to the five indicator questions significantly increased by 5–20.6%.

**Conclusion:**

Teachers’ knowledge of TDI was inadequate yet can improve with online education. Efforts among dental professionals, the media, and targeted interventions will ensure adequate knowledge while also improving children’s dental health.

## Introduction

One of the most common areas of paediatric emergency care is paediatric dental traumatology. Different injuries occur daily with characteristic temporal variability. The injuries to children’s teeth can occur for a variety of reasons with seasonal and age-related variability in the causes and manner of injuries. It is essential to provide emergent care following an injury because prompt, adequate care is paramount to the proper healing of oral injuries. Adequate management of traumatised teeth is essential because periodontal and pulpal tissue injuries increase the risk of bacterial invasion and secondary infection (Trope 2002). On the other hand, an Iraqi study by Yassen G. et al. reported that only 5% of the 294 children surveyed experienced access to a professional dentist within 24 h, whereas most of the children were treated after one month. Education of parents and teachers, including prompt care, is obviously desirable in all cases (Yassen et al. 2013).

Traumatic dental injuries (TDI) are common, and the estimated prevalence of permanent detention worldwide is 15.2%, according to a recent meta-analysis. The authors conclude that 85% of orofacial injuries cause trauma in the dentition, and the incidence by preschool children may rise to 17% (Petti et al. 2018). A different survey of children aged 6–12 showed that 55% of injuries occur outdoors, whereas nearly 40% occur during autumn (Yassen et al. 2013). Typical locations are limited to physical education classes and training sessions in schools, and rarely in other areas in the institution, such as classrooms and corridors (Oikarinen and Kassila 1987). Data from many countries showed that one-third of all preschool children suffered traumatic dental injuries involving the primary dentition, one-fourth of all school children, and nearly one-third of adults have suffered trauma to permanent dentition (Zaleckiene et al. 2014). Traumatic events occur with highest frequency at schools, home, sports facilities, or in streets and roadways (Cagetti et al. 2019), with approximately one-third of the injuries occurring in a school environment and another one-third at home during school, with boys more likely to be injured than girls (Oikarinen and Kassila 1987). Additionally, disabled children and behaviour-challenged children are injured more than their classmates (Pani et al. 2014). The consequences of deciduous dental injuries may have a major impact on the physical and mental health of children. The outcome is dependent on the injury type and the time span between the injury and treatment. Severe consequences include inflammation, pulp tissue necrosis, ankylosis, and potential impact on permanent tooth development. The incidence of TDIs was observed in 4.2–35% of various age groups, and the permanent effect was measured at approximately 20–30% of the injured (Alhaddad et al. 2019). Published studies identify a gap in the dental knowledge of adult population, including teachers, emphasising the role of health professionals in filling this gap with proper education in the field of TDIs.

Dental knowledge and behaviour regarding first aid among teachers are crucial in the management of school injuries. Teachers must be well educated and thoroughly conversant in the management of traumatic dental injuries. Based on published literature, the authors hypothesise that a limited number of indicator questions can be used to estimate general knowledge among educators regarding TDI management. Furthermore, these questions provide the key points for developing professional guidelines for teachers in the first-aid management of dental injuries.

There have been no studies analysing dental injuries in Hungary or describing the knowledge among schoolteachers regarding the first-aid management of dental injuries. Thus, the aim of our study was to measure dental care competencies among Hungarian educators and to identify gaps in knowledge, as a result providing the basis for upcoming educational campaigns. An additional objective was to evaluate the learning outcomes (five key questions about first-aid management of dental injuries) of an informative handout regarding TDI management for schoolteachers by comparing teachers’ knowledge prior to (pre-intervention) and following the dissemination of the educational content (post-intervention).

## Materials and methods

### Study design

This cross-sectional observational national study was conducted with the participation of kindergarten teachers (< 6 years), primary (6–10 years) and elementary (10–14 years children) school educators, and secondary school teachers working with children above 14 years of age. The voluntary self-administered online questionnaire assured anonymity to all respondents and was approved by the Regional Committee for Research Ethics, University of Pécs. Following professional evaluation of the questionnaire responses, an informative handout including infographics and educational content was developed targeting schoolteachers with a focus on the lack of knowledge identified by the five indicator questions.

Comprehensibility was improved by avoiding professional jargon and by including infographics. Additionally, further educational material was created and illustrated with short informative videos for three specific stakeholder groups: children, parents, and schoolteachers. These online info-packages were made accessible on a dedicated webpage of Colgate Palmolive Hungary. In March 2021, teachers’ knowledge was re-evaluated using our original 15-item questionnaire. To illustrate the effectiveness of an educational intervention for TDI management, teachers were asked to read the content of the educational material provided as a PDF attachment of the e-mail message and link to the online info-package and complete the Q15 questionnaire afterwards.

### The questionnaire

A pilot questionnaire including 13 single-choice questions was developed based on the publication of Chandukutty D. et al. (Chandukutty 2017) to determine the details of dental knowledge related to TDI. This 13-item paper-based pilot questionnaire (Q13) consisted of two sets of questions: sociodemographic data and experience and professional knowledge regarding TDI. This pilot Q13 was spread out on a paper base, utilising professional contacts, and 289 responses were collected. Based on the pilot responses, the authors revised the list of questions, including the addition of two new questions (sport teacher, self-evaluation). The final Q15 study questionnaire was reviewed and assessed by an academic practitioner. Subsequently, this 15-item national language anonymous online questionnaire was distributed via e-mail to 2720 educational institutions’ public email addresses available from the Hungarian Educational Authority website. Responses were collected from January 2019 through May 2019. Based on the clinical experience of the authors and evidence-based treatment guidelines for dental trauma (University Hospital of Copenhagen 2022), five indicator questions No q8-q12 (see Table 2) from our survey were selected to evaluate the appropriateness regarding the general knowledge among schoolteachers and their perceptions in reference to TDI. The English translation of the Q15 questionnaire is available as an Appendix.

### Statistical analysis

Statistical analysis was performed using IBM SPSS Statistics 28 Software. Pre- and post-intervention respondent categorical data are presented in numbers and percentages. Descriptive data were compared using the chi-square or Fischer’s exact test and the Mann–Whitney *U*-test. Statistical significance was established as a *p*-value of < 0.05.

## Results

### Respondent characteristics, experience, and perceptions of TDI

In 2019, 1426 respondents completed the initial pre-intervention survey. According to the Hungarian Central Statistical Office, 149,362 teachers are employed in Hungary (Hungarian Central Statistical Office 2021); therefore, our sample represents nearly 1.3% of the total teacher population throughout Hungary. Most respondents were female, nearly half of whom were aged over 51 years, in which age distribution adheres to the demographical characteristics of teachers throughout Hungary. Geographical distribution, according to population size and density, accurately reflects Hungary because one-fifth of the respondents live in the capital city and less than half live in rural areas. The relatively even distribution of the respondents based on educational institutions was favourable, providing representative results from all age groups in childhood; however, physical education teachers were somewhat overrepresented in the study population. See the respondent characteristics in Table 1.

**Table 1:** Respondent characteristics and experiences regarding TDI in 2019 (*n* = 1426)

Characteristics and question topics	*n*	%
q1. Gender		
Female	1273	89.3
Male	153	10.7
q2. Age (years)		
20–40	274	19.2
41–50	502	35.2
51 and above	650	45.6
q3. Practice setting (inhabitants)		
Village (< 5000)	305	21.4
Town (5–20,000)	319	22.4
City (20–100,000)	211	14.8
Large city (100,000 -1 million)	255	17.9
Capitol (> 1 million)	336	23.5
q4. Institution and age of the children		
Kindergarten (< 6 years)	380	26.6
Primary school (6–10 years)	377	26.4
Elementary school (10–14 years)	345	24.2
Secondary school (> 14 years)	324	22.7
q5. Previously eyewitness of TDI in children	740	51.9
q6. Teeth are most frequently traumatized		
Lower frontal teeth	45	3.2
Upper frontal teeth	1369	96.0
Molar teeth	12	0.8
q7. Age group of children at the highest risk of TDIs		
1–3 years	116	8.1
4–6 ears	527	39.9
7–10 years	637	44.7
11–15 years	132	9.3
15–18 years	14	1.0
q13. Activity perceived as the most dangerous to children’s tooth integrity		
Ballgames (e.g. soccer)	193	13.5
Winter sports	105	7.4
Balance-bike	305	21.4
Playground games	823	57.7
q14. Physical Education Teacher	427	29.9
q15. Respondents who consider themselves sufficiently informed regarding the care of dental accidents and injury	193	13.5

Although more than half (51.9%) of the respondents had previously witnessed TDI, less than one out of six educators (13.5%) in our sample considered themselves sufficiently informed regarding TDI management. Most respondents have appropriate knowledge in reference to the most frequent location of dental injuries, since 96% responded correctly in which the upper front teeth are most frequently affected. The majority (82.3%) of kindergarten educators chose the age group under 6 years, while 63.9% of secondary school teachers chose the age group older than 6 years (*p* < 0.001). Playground games are considered the most dangerous activities to children’s teeth integrity by most (57.7%) respondents, somewhat more so among physical education teachers who consider balance-bike riding as one of the most dangerous activities when compared with non-sport conductors (27.9% vs. 18.6%). Less respondents who conduct physical education (7.7%) perceive ballgames to be dangerous than others (16.0%) (*p* < 0.001).

### Evaluation of teachers’ knowledge regarding dental injuries

Indicator questions q8–12 were analysed to evaluate the TDI-specific knowledge of respondents in the initial dataset (**see **Table 2**.**). Most teachers will immediately refer the child to a dentist (51.3%) or as soon as possible (35.6%), specifically in the case of bleeding originating from the oral cavity. In consideration of the urgency of dental treatment, we found that 40.3% preferred the shortest time, 31.5% considered 6 h enough, while a relatively high ratio of inappropriate (17.7%) and ‘I don’t know’ (10.5%) responses exhibited uncertainty among respondents regarding this crucial question. Accordingly, considering the urgency of taking the child to a dental professional, 71.8% of educators responded correctly. Most schoolteachers (79.0%) will contact parents after witnessing a dental trauma or immediately notify a dentist (13.0%) following an injury. However, some (6.3%) will manage the injury and continue the educational activities. A large majority (81.3%) of the participants correctly responded that the avulsed teeth must be taken to the dentist in an appropriate medium.

**Table 2:** Teachers’ knowledge regarding dental injuries based on five indicator questions in 2019 (*n* = 1426)

Questions on TDI knowledge	*N*	%
q8. “What will you do in the case of bleeding originating from the oral cavity following TDI?”		
Try to stop bleeding using tissue paper	158	11.1%
I will immediately refer the child to a dentist	508	35.6%
Apply mouth rinse to the tooth and refer the child to the dentist as soon as possible	731	51.3%
If the bleeding soon stops, it is enough to visit the dentist within the span of two weeks	29	2.0%
q9. “How urgent should the injury be managed?”		
Within 30 min	574	40.3%
Within 6 h	449	31.8%
Any time during the day	253	17.7%
I don’t know	150	10.5%
q10. “What is the first step in the case of oral injury?”		
Contact parents and advise them to take the child to a clinic	1126	79.0%
Manage the injury of the child and continue education/class	90	6.3%
Immediately notify a dentist	185	13%
I don’t know what to do	25	1.7%
q11. “What is the appropriate management of an avulsed tooth?”		
Tooth should be replaced immediately	45	3.2%
There is no point in replacing it	116	8.1%
It should be taken to the dentist in an appropriate medium	1159	81.3%
I don’t know	106	7.4%
q12. “How should the avulsed tooth be cleaned”		
Scrub the tooth gently using a toothbrush	238	16.7%
Rinse with saline or under tap water	802	56.2%
Rinse with household antiseptic	1	0.07%
Putting the tooth back into the socket without cleaning	99	6.9%
Not necessary to clean	286	20.0%

^*^Appropriate answers are underlined

Respondent characteristics (including age, practise setting, institution) have correlated with indicator questions to identify the segment of the teacher population with the lowest preparedness regarding TDI management. Practice settings and institution did not seem to correlate with TDI management (*p* > 0.05). We introduced several illustrative examples demonstrating the significance of the knowledge and management of traumatic dental injuries among schoolteachers. In the case of bleeding originating from the oral cavity, 61.8% of respondents older than 60 years will immediately transport the injured child with TDI to the dentist, whereas only 41.2% under the age of 40 years will react the same. Thus, the tendency to take immediate action seemingly correlates with age (*p* < 0.001). The urgency of TDI management does not substantially differ when teachers previously witnessed an injury; at the same time, a significantly larger number of respondents considering themselves sufficiently informed would manage the injury within 30 min (50.3%) versus the individuals who feel unprepared (38.7%) (*p* < 0.001). Regarding the first step following an injury, the responses showed correlation with gender, in which more female teachers (80.1%) will contact parents and advise them to take the child to a clinic than male teachers (69.3%). Furthermore, 3.9% of male teachers responded, “I do not know,” while only 1.5% of female teachers offered the same response (Chi^2^ test, *p* = 0.002). A similar relationship was observed when the appropriate management of the avulsed tooth was evaluated, in which a larger number of male respondents perceived replacing the avulsed tooth as pointless than when compared with their female counterparts (18.3% versus 6.9%), while more female teachers (82.8%) transferred the avulsed tooth to the dentist in an appropriate medium than when compared with male respondents (68.6%) (*p* < 0.01). Consequently, teachers’ preparedness regarding specific TDI management questions implies a distinctive correlation with gender.

### Effectiveness of educational intervention

Learning material (in PDF, and website online) developed by the authors was disseminated to the previously used electronic address list. Ten months following this educational intervention, the knowledge among schoolteachers was re-evaluated using the same Q15 online questionnaire form. Since the Colgate Palmolive website offered a game option in addition to learning the information material, it was assumed that the same teachers who had studied the information material and participated in the game had completed the questionnaire. This assumption was supported by the fact that the post-intervention series of responders exhibited similar demographical data. Although a smaller number of responses were obtained, the respondent sample size (*n* = 622) was still considered representative. This post-intervention dataset did not significantly differ in gender (*p* = 0.85), age category (*p* = 0.42), teachers of physical education (*p* = 0.48) or status in being an eyewitness of a TDI (*p* = 0.12). The distribution of respondents significantly differed based on the practice settings (*p* < 0.001), since a somewhat higher number of respondents completed the post-intervention questionnaire from less populated areas. Furthermore, the two datasets differ (*p* = 0.003) based on institution type. Consequently, the age of children taught, in ratio of schoolteachers employed in kindergartens (< 6 years) and primary schools (6–10 years), was higher in the post-intervention sample.

**Fig. 1:**
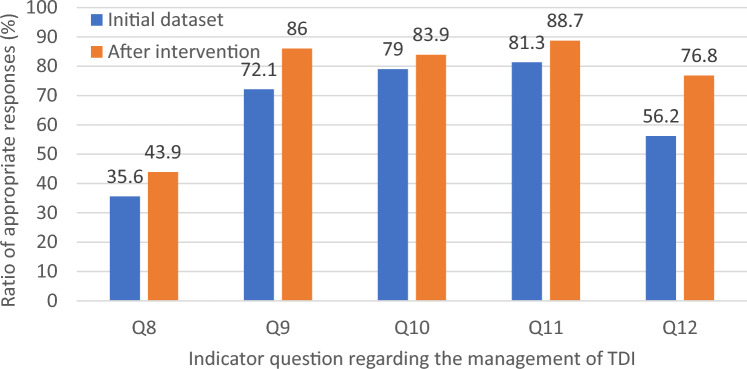
(alternative to Table 3): Relative change in appropriate answers for five indicator questions regarding the management of TDI among schoolteachers before and following educational intervention (initial pre-intervention dataset *n* = 1426 and post-intervention responses *n* = 622)

The indicator questions were designed to measure good answers in absolute terms: different questions have different numbers of good answers. In q8, a specific event (bleeding) needs to be addressed, which makes snap decisions difficult. Therefore, the set of answers can exercise increased diversity. Elsewhere, we measured lower levels of knowledge gaps, yet the relatively high number of ‘don’t know’ responses also indicated significant gaps in responsiveness and skills levels. Although the participants are not dentally educated individuals, in consideration of this study, a perfect answer cannot be expected. As seen in Table 3, the percentage of appropriate responses to one but all indicator questions regarding TDI management significantly increased by 5%–20.6% following our intervention. The intervention significantly increased participants’ evaluation of self-knowledge, since the ratio of respondents considering themselves sufficiently informed underwent a substantial increase, from 13.5 to 30.2% following the educational intervention (Chi^2^ test, *p* < 0.001) (Fig. 1).

**Table 3:** Relative change in appropriate answers for five indicator questions regarding the management of TDI among schoolteachers following educational intervention (pre-intervention initial dataset *n* = 1426 and post-intervention responses *n* = 622)

Question topic	Ratio of appropriate responses (%)		Relative change (%)	*P*-value
	Pre-intervention initial dataset (*n* = 1426)	Dataset following the educational intervention (*n* = 622)		
q8. What will you do in the case of bleeding originating from the oral cavity following TDI?	35.6	43.9	+ 8.3	< 0.001
q9. How urgent should the injury be managed?	72.1	86.0	+ 13.9	< 0.001
q10. What is the first step in the treatment of oral injury?	79.0	83.9	+ 5.0	0.009
q11. What is the appropriate management of an avulsed tooth?	81.3	88.7	+ 7.5	< 0.001
q12. How should the avulsed tooth be cleaned?	56.2	76.8	+ 20.6	< 0.001

## Discussion

A lack of knowledge regarding TDI and failure to provide first aid may yield unfavourable consequences, including pain, loss of function, and aesthetic problems, resulting in physical, emotional, and social consequences for the affected children and their families (Borges et al. 2017; Cagetti et al. 2019). In our study, we recognised knowledge gaps and identify groups of teachers associated with a general knowledge deficit. A specified, targeted learning opportunity was created for schoolteachers to update their knowledge of TDI management.

One of the most interesting responses was the self-evaluation of responders, since a wide number of educators admitted the lack of knowledge related to the topic. Additionally, respondents also had to cope with a lack of self-esteem. It is a psychological fact that the lower the level of self-esteem, the more difficult it is to acquire new knowledge. It is imperative that we highlight that the knowledge of teachers with a demonstrated gap needs to be improved. Although the level of self-esteem among teachers increased by 20% following our intervention programme, its further development is clearly needed.

It was assumed that anyone who had previously witnessed a TDI has a different perception of the accident due to the event. Interestingly, when we compared the institutions of teachers’ employment and the most endangered age group of children, we found that teachers tend to consider their level of education as the most vulnerable age group. We identified substantial shortcomings in the management of avulsed teeth. The appropriate medium or transport fluid in which the avulsed tooth (and especially the periodontal cells) can be used to transfer the tooth to the dentist is also crucial for the favourable prognosis of avulsed teeth. When questioned on the recommended washing method, only somewhat more than half of the responding schoolteachers (56.2%) gave the correct response. Nevertheless, the remainder of the respondents did not know which rinsing agent was the best medium, as other options listed may decrease the success rate of replantation of the tooth.

Similar studies have been conducted in several countries (Bayrak et al. 2012; Blakytny et al. 2001; Feldens et al. 2010; Glendor 2009). Our results are aligned with those of earlier studies because there is a general lack of TDI treatment knowledge among teachers. Knowledge and skills regarding the management of TDI can be developed if the relevant education channels are adequately targeted. In relation to the study, Hungarian teachers reflected how they welcome further training on this topic, since they had not received any relevant information either during their university education or afterwards. Based on similar studies in different countries, including Croatia (Bakarčić et al. 2017) and Singapore (Sae-Lim and Lim 2001), we assume that teachers knew how to react, yet without previous experience and specific training, they do not apply their knowledge.

The next problem was the inappropriate management of traumatized teeth. The subject may know what to do, yet due to differences in individual, social, cultural, ethical, and ethnic factors, they fail to perform the appropriate task. Unfortunately, this is a typical behaviour among adults worldwide, and not limited to teachers alone. Elimination of such behaviour is an essential goal of preventive medicine.

Additionally, our aim was to create and maintain targeted dental education that supports children, teachers, school nurses, and other non-specified personnel. We created this information package to support the study. A similar study was conducted in Poland, in which, following targeted instruction, significantly better results were detected (Baginska and Wilczynska-Borawska 2012).

Based on the experience gained during this study, our team published an educational tool in the form of a poster entitled “Save Your Teeth”, originally developed by the International Association of Dental Traumatology (IADT). The IADT accepted our Hungarian version representative of the document, and the electronic version was published on numerous websites nationwide. The effect of this poster has been supported by previously published international literature references (Lieger et al. 2009; Walker and Brenchley 2000), since teachers who have seen the poster responded significantly better when compared with colleagues who had no previous exposure to the poster’s information.

Our study has several strengths; however, it also has numerous limitations. It must be noted that physical education teachers are overrepresented in our study sample. Although gender and age distribution was not representative of the general Hungarian population, our study population represented Hungarian schoolteachers according to national demographics. Admittedly, the initial (pre-intervention) dataset of respondents and the post-interventional cohort differ in size; however, demographics are comparable, and we are aware that evidence-based and real-world efficacy of the applied educational programme cannot be directly measured. Additionally, we were not able to evaluate teachers’ knowledge in both pre- and post-intervention datasets as the questionnaire was made anonymous and we did not collect individual identifiers. Less responses in the second dataset may originate from a lack of interest in filling the same questionnaire and change in attitudes towards online surveys of e-mail communication following the pandemic. Nevertheless, considering the favourable increase in awareness and knowledge, our proposed educational method may provide a promising solution to identify and fill knowledge gaps in positively impacting schoolteacher responses to effective TDI management.

## Conclusion

Most teachers are uninformed, and the results easily demonstrate that educators also find themselves undereducated regarding TDI management. Furthermore, there is a need among schoolteachers to develop their knowledge in the field of dental injury management. In reviewing the results of the five most important and informative indicator questions, one can easily see a significant change in the knowledge of respondents following the educational intervention. Because the long-term efficacy of preventive interventions is difficult or nearly impossible to measure, we encourage the scientific community to conduct similar studies globally and facilitate similar educational campaigns at the national level.

## Data Availability

The data supporting the findings of this study are available on request from the corresponding author.

## Questionnaire tool for the evaluation of knowledge and management of traumatic dental injuries of schoolteachers

(English translation of the Hungarian questions used in the study)

q1. “Your Gender?”

female

male

q2. “Your age (years)?”

q3. “What size municipality do you work in?”

village (< 5000)

town (5-20,000)

city (20-100,000)

large city (100,000 -1 million)

capitol (>1 million)

q4. “What type of institution do you work in?”

kindergarten (<6 years)

primary school (6-10 years)

elementary school (10-14 years)

secondary school (>14 years)

q5. “Have you ever been an eyewitness of any dental injuries in children?„

yes

no

q6. “Which teeth are most frequently traumatized?”

lower frontal teeth

upper frontal teeth

molar teeth

q7. “Which age-group of children are at the highest risk for dental injuries?”

1-3 years

4-6 ears

7-10 years

11-15 years

q8. “What will you do in the case of bleeding originating from the oral cavity following dental injury?”

Try to stop bleeding using a tissue paper

I would immediately refer the child to a dentist

Apply mouth rinse to the tooth and refer the child to the dentist as soon as possible

if the bleeding soon stops, it is enough to visit the dentist within the span of two weeks

q9. “How urgent should the injury be managed?”

within 30 minutes

within 6 hours

any time during the day

I don’t know

q10. “What is the first step in case of oral injury?”

Contact parents and advise them to take the child to a clinic

Manage the injury of the child and continue education/class

Immediately notify a dentist

I don’t know what to do

q11. “What is the appropriate management of the avulsed tooth?”

The tooth should be replaced immediately

There is no point in replacing it

It should be taken to the dentist in an appropriate medium

I don’t know

q12. “How should the avulsed tooth to be cleaned”

Scrub the tooth gently using a toothbrush

Rinse it with saline or under tap water

Rinse it with household antiseptic

Put the tooth back into the socket without cleaning

Not necessary to clean

q13. “Which activity is the most dangerous to children’s teeth integrity?”

ball games

winter sports

balance-bike

playground.

q14. “Do you conduct physical education in the school?”

yes

no

q15. “Do you find yourself sufficiently informed about regarding the care of dental accidents and injury?”

yes

no

## Appendix: English translation of the handout

Injuries to teeth and surrounding tissues are very common in school accidents. Injuries usually involve a lot of pain and bleeding, often combined with fractures of the surrounding bones. In 80–90% of cases, the upper front teeth are damaged, and the most at risk age group is elementary classes 5-8th class. Different risk factors are encountered according to age, with young children are more likely to be injured on the playground, while at older ages sports are more likely to be the injury risks. If an accident does occur, it is the responsibility of the adults present at school to target the action, as a trained helper can significantly improve the recovery process. The first thing to do is always to inform the guardian of the child, and in the case of suspected negligence, the authorities should also be informed. In the meantime, of course, the child and the injury must be monitored. It must be made clear whether the child is oriented in space and time, whether he or she can respond to the questions asked. If bleeding is heavy, it can be stopped for a short time with mild pressure, but presumably the bleeding will soon stop on its own, it is more important to gently disinfecting and covering the area and preferably seeing a dentist immediately. General guideline, that the child should be treated within 6 h of the injury. If a tooth is visibly out, it should be gently rinsed with tap water or saline solution and transported without cleaning in an appropriate liquid (milk, saliva, mild saline) to the dentist.
